# Chemical sedation of excited delirium in the pre-hospital setting

**DOI:** 10.29045/14784726.2020.12.4.4.34

**Published:** 2020-03-01

**Authors:** Richard Armour

**Affiliations:** British Columbia Emergency Health Services; University of Sheffield: ORCID iD: https://orcid.org/0000-0001-7528-961X

**Keywords:** delirium, paramedic, sedation

## Abstract

A 30-year-old male presents to emergency medical services profoundly combative with a Richmond Agitation–Sedation Scale of +4 after reported use of intravenous methamphetamines. A preliminary diagnosis of excited delirium syndrome is made based on the history obtained and the decision is made to chemically sedate the patient. While preparing for sedation, you wonder which pharmacological agent will produce the fastest and safest sedation in this patient population.

## Clinical scenario

A 30-year-old male presents to emergency medical services (EMS) profoundly combative with a Richmond Agitation–Sedation Scale of +4 after reported use of intravenous methamphetamines. A preliminary diagnosis of excited delirium syndrome (ExDS) is made based on the history obtained and the decision is made to chemically sedate the patient. While preparing for sedation, you wonder which pharmacological agent will produce the fastest and safest sedation in this patient population.

## Clinical question

In patients presenting with ExDS in the pre-hospital environment, is the use of benzodiazepines, antipsychotics or ketamine superior in producing rapid and uncomplicated sedation?

## Search strategy

*United States of America National Library of Medicine (up to September 2019):*

((Excited Delirium[Title/Abstract] OR ExDS[Title/Abstract] OR Agitation[Title/Abstract] OR Acute Behavioural Disturbance[Title/Abstract])) AND (Prehospital[Title/Abstract] OR pre-hospital[Title/Abstract] OR paramedic[Title/Abstract] OR EMS[Title/Abstract] OR Emergency Medical Services[Title/Abstract])

*Cumulative Index to Nursing and Allied Health Literature (up to September 2019):*

TX (paramedic or ems or emergency medical service or prehospital or pre-hospital or ambulance or emergency medical technician or emt) AND TX (excited delirium syndrome OR ExDS OR agitation OR acute behavioural disturbance)

Literature was considered relevant if it described a piece of primary research in the pre-hospital setting, with a focus on the pharmacological management of ExDS. Literature was excluded if the research was undertaken in a hospital-based setting, or if it was non-interventional in nature.

## Search results

*United States of America National Library of Medicine:*

90 results, of which nine were considered relevant.

*Cumulative Index to Nursing and Allied Health Literature:*

145 results, of which eight were considered relevant. None in addition to those identified previously.

## Relevant literature

See Table 1 for relevant literature.

**Table 1.** Relevant literature.

Reference	Pharmacology	Inclusion	Study design	Outcomes	Results	Weaknesses
**Rosen et al. (1997)**	Droperidol 5 mg vs. placebo	Convenience sample of adult males transported to single hospital emergency department, described as ‘combative’ by paramedics.	Single-blind, randomised controlled trial	Reduction in agitation	71% reduction in agitation at 10 mins (p < 0.001) in intervention group	Convenience sample with illogical inclusion and exclusion criteria, particularly considering the small volume system the trial was undertaken in. Unvalidated sedation scale used to assess level of agitation. Over half of the patients in the placebo group never required sedation, bringing the internal validity into question. Unclear whether outcome assessors were blind to allocation.
				Requirement for further sedation	48% of patients in placebo group required further sedation, against 13% in the droperidol group (p = 0.01)	
				Adverse reactions	One patient in droperidol group developed akathisia	
**Ho et al. (2013)**	Ketamine 500 mg and ketamine 375 mg	One 35-year-old male and one 40-year-old female.	Retrospective case series	Time-to-sedation	4 mins and 3 mins respectively	No comparator population. Poorly described objectives from the case series. Unclear why ketamine was chosen over standard therapies for the EMS agency (enrolment bias).
**Scheppke, Braghiroli, Shalaby, & Chait (2014)**	Ketamine 4 mg/kg with subsequent midazolam 2 mg	Patients treated according to EMS agency’s excited delirium protocol.	Retrospective case series	Time-to-sedation	Mean 2 mins (range 1–5 mins)	No comparator population. Significant protocol deviation, not all patients received the prescribed subsequent dosage of midazolam with no sub-group analysis performed. Significant amount of missing data from patient records, with total reliance on this incomplete data for recording of adverse events (interpretation bias). No indication of how records were identified within the EMS system, leading to potential inclusion bias. No objective measure of sedation reported.
				Depth of sedation	‘Adequate’ sedation achieved in 50 of 52 patients	
				Haemodynamic or respiratory events	Three cases of significant respiratory events noted, no haemodynamic events	
**Isenberg & Jacobs (2015)**	Midazolam 5 mg vs. haloperidol 5 mg	Patients treated according to EMS agency’s protocol for ‘behavioural disturbances and psychiatric emergencies’.	Open-label randomised controlled trial	Times to RASS +1 or less	In patients receiving haloperidol, mean time to RASS of less than +1 was 24.8 mins (95% CI 8–49 mins), while in patients receiving midazolam, mean time to RASS less than +1 was 13.5 mins (95% CI 8–19 mins)	Small sample size (five in each arm of the trial). Paramedics were not blinded to allocation, as it was known to paramedics that patients were allocated according to day of the month. Single-centre trial with small volume of patients, never likely to enrol required numbers. Although patients were assessed with the RASS score, they were enrolled based on protocol definitions, limiting external validity. Five additional patients excluded by the EMS agency medical control with no reason given.
				Requirement for additional sedation	Two patients in the haloperidol group required additional sedation, compared to no patients in the midazolam group	
				Adverse events	None in either group	
**Keseg, Cortez, Rund, & Caterino (2015)**	Ketamine 4 mg/kg	All adult patients treated with ketamine by single EMS agency.	Retrospective case series	Patients recorded as ‘improved’ on patient report form	Improvement recorded in 91% of cases (95% CI 77–98%)	No comparator group. Small sample size (32 examined). Review of clinical outcomes was not blinded to intervention. Incomplete data present for a number of patients. Subjective outcome as primary outcome. Poor analysis of patients requiring endotracheal intubation.
				Requirement for additional sedation	40% of cases required additional sedation (95% CI 24–58%)	
				Requirement for endotracheal intubation	23% of cases required endotracheal intubation (95% CI 10–40%)	
**Cole et al. (2016)**	Haloperidol 10 mg vs. ketamine 5 mg/kg	Adult patients with an AMSS of +2 or +3 transported to a single hospital.	Prospective observational study	Time to AMSS score of less than +1	Ketamine produced an AMSS score of less than +1, 12 mins faster than haloperidol (p < 0.0001, 95% CI 9–15 mins)	Excluded patients with extreme agitation. Unblinded, non-randomised study design. Patients were allocated to treatment based on season. EMS agency serves multiple hospitals, with patients only enrolled if they were to attend a single site. Unclear why this was the chosen study design.
				Depth of sedation	95% of patients in the ketamine group achieved adequate sedation, compared with only 65% in the haloperidol group (p < 0.0001)	
				Adverse events	49% of patients in the ketamine group experienced an adverse event against 5% in the haloperidol group (p < 0.0001, 95% CI 30–57%)	
**Cole et al. (2018)**	Ketamine 5 mg/kg	Patients with active physical violence to themselves or others and an AMSS of +4.	Retrospective pre- and post-analysis	Time to AMSS score of less than +1	Median time to sedation was 4.2 mins (95% CI 2.5–5.9 mins)	EMS agency serves multiple hospitals, which meant that only 56 of 158 potentially eligible patients were analysed. Rates of complications much higher than in other published literature, raising questions regarding external validity of findings. No comparator group.
				Depth of sedation	AMSS of less than +1 achieved in 90% of cases	
				Adverse events	Complications occurred in up to 57% of patients, with intubation, hypersalivation, vomiting and emergence phenomena common	
**Page et al. (2018)**	Midazolam 5 mg vs. droperidol 10 mg	Patients adjudged to have an acute behavioural disturbance as primary complaint and an SAT of 2 or greater.	Controlled before-and-after study	Time to reduction in SAT by 2 or more	Median time to sedation in droperidol group was 22 mins (IQR 16–35 mins), compared to 30 mins for midazolam (IQR 20–44 mins)	Small enrolment bias (7% missed cases). Higher proportion of SAT score of 3 in the midazolam group, with possible confounding.
				Requirement for additional sedation	Additional sedation required in 4% of patients receiving droperidol (95% CI 1–9%), compared to 14% of patients receiving midazolam (95% CI 9–21%)	
				Adverse events	16% higher rate of adverse events in the midazolam group compared with the droperidol group (95% CI 8–24%, p = 0.0001)	
**O’Connor et al. (2019)**	Ketamine 4 mg/kg vs. haloperidol 5 mg and midazolam or lorazepam 4 mg	All adult patients treated for combative or agitated behaviour.	Retrospective pre- and post-analysis	Requirement for endotracheal intubation	More frequent intubation with use of ketamine (OR 8.77, 95% CI 1.10–69.68)	Seven missed patients because of transport to alternative site. No objective measurement of agitation or combativeness, making it difficult to analyse requirements and improvement with sedation. During ketamine period, haloperidol and midazolam remained available for use and 27 patients received this in place of ketamine, potentially suggesting selection bias. 55% of intubations were performed by a single provider, with potential for provider discomfort with ketamine influencing intubation rate. Staff injury not uniformly documented and may be influenced by reporting bias.
				Hospital admission	No difference in requirements for admission (OR1.97, 95% CI 0.84–4.61)	
				Additional restraint (any)	Additional restraint required more often in the ketamine group (OR 2.19, 95% CI 1.15–4.15)	
				Additional restraint (chemical)	Additional pharmacology required more often in ketamine group (OR 2.94, 95% CI 1.49–5.80)	
				Staff injury	No difference between groups (OR 1.94, 95% CI 0.71–5.28)	

Note: AMSS = Altered Mental Status Scale; CI = Confidence Interval; EMS = Emergency Medical Services; IQR = Interquartile Range; RASS = Richmond Agitation–Sedation Scale; SAT = Sedation Assessment Tool.

## Comment

Despite the challenges which patients suffering from ExDS will present to practitioners, literature examining the management of these patients in the pre-hospital environment is exclusively from the United States or founded in the setting of an emergency department with regional variations in the optimal sedative recommended for use in this patient population. Although the pre-hospital and emergency department settings share a number of features, the pre-hospital environment is an unpredictable, profoundly resource-poor setting without consistent access to anaesthesia and advanced medical therapies potentially required for management of deteriorating patients with ExDS. This is of particular importance when considering not simply the efficacy, but the safety of pharmacological management of patients presenting with ExDS.

## Clinical bottom line

There is insufficient evidence to recommend one pharmacological agent in the management of ExDS within the pre-hospital environment generally, and no evidence specific to specialist paramedic practice in the United Kingdom. Although ketamine is associated with the most rapid onset of adequate sedation in patients with ExDS, it is also associated with the highest rate of adverse events and its use is frequently reported to result in the requirement for endotracheal intubation. However, it is unclear whether the requirement for intubation is driven by clinical need or provider discomfort, given the number of intubations performed by single providers within multiple studies. Antipsychotic agents such as haloperidol and droperidol appear to have a superior safety profile when compared against ketamine and midazolam, although they are associated with a protracted time to adequate sedation, which is of particular relevance in the setting of an ExDS patient and in the setting of the resource-poor pre-hospital environment.

The choice of pharmacological agent for sedation of the patient with ExDS will depend on the local legality of paramedic-led administration of scheduled medications, availability, clinician comfort and available resources to manage unexpected complications. Further high-quality controlled trials of benzodiazepines, antipsychotics and ketamine are required before any recommendation for widespread use of any agent can be made.

## Conflict of interest

None declared.

## Funding

None.
